# Retrospective efficacy analysis of immune checkpoint inhibitors in patients with EGFR‐mutated non‐small cell lung cancer

**DOI:** 10.1002/cam4.2037

**Published:** 2019-02-21

**Authors:** Tadaaki Yamada, Soichi Hirai, Yuki Katayama, Akihiro Yoshimura, Shinsuke Shiotsu, Satoshi Watanabe, Toshiaki Kikuchi, Kazuki Hirose, Yutaka Kubota, Yusuke Chihara, Taishi Harada, Keiko Tanimura, Takayuki Takeda, Nobuyo Tamiya, Yoshiko Kaneko, Junji Uchino, Koichi Takayama

**Affiliations:** ^1^ Department of Pulmonary Medicine, Graduate School of Medical Science Kyoto Prefectural University of Medicine Kyoto Japan; ^2^ Department of Respiratory Medicine Japanese Red Cross Kyoto Daiichi Hospital Kyoto Japan; ^3^ Department of Respiratory Medicine and Infectious Diseases Niigata University Graduate School of Medical and Dental Sciences Niigata Japan; ^4^ Department of Respiratory Medicine Japanese Red Cross Kyoto Daini Hospital Kyoto Japan; ^5^ Department of Medical Oncology Fukuchiyama City Hospital Fukuchiyama, Kyoto Japan; ^6^ Department of Respiratory Medicine Uji‐Tokushukai Medical Center Uji, Kyoto Japan

**Keywords:** biomarker, EGFR mutation, immunology, non‐small cell lung cancer

## Abstract

**Background:**

Treatment with epidermal growth factor receptor (EGFR)‐tyrosine kinase inhibitors (TKIs) leads to initial response in most patients with *EGFR*‐mutated non‐small cell lung cancer (NSCLC). In contrast, little is known of the subpopulation of patients with NSCLC with *EGFR* mutations who exhibit clinical outcomes that require treatment with immune checkpoint inhibitors (ICIs). Therefore, to identify eligible cases to treat with ICIs, we retrospectively analyzed the correlation between clinical features and the efficacy of ICIs in patients with *EGFR* mutations.

**Patients and Methods:**

We retrospectively analyzed patients with advanced NSCLC harboring *EGFR* mutations who were treated with ICIs after developing resistance to EGFR‐TKIs between February 2016 and April 2018 at 6 institutions in Japan. The association between clinical outcomes and the efficacy of ICIs was investigated.

**Results:**

We enrolled 27 patients who harbored *EGFR*‐activating mutations. The objective response and disease control rates were higher in patients with uncommon *EGFR* mutations than in those with common *EGFR* mutations (71% vs 35.7% and 57% vs 7%, *P* = 0.14 and *P* < 0.01, respectively). Patients with uncommon *EGFR* mutations or without T790M mutations exhibited a significantly longer median progression‐free survival than those with common *EGFR* mutations or with T790M mutations (*P* = 0.003 and *P* = 0.03, respectively).

**Conclusion:**

Patients with uncommon *EGFR* mutations and without T790M mutations are associated with the best outcomes for treatment with immunotherapy among those with *EGFR*‐mutated NSCLC, based on retrospective analysis. Further research is needed to validate the clinical biomarkers involved in ICI responders with *EGFR* mutations.

## 1 INTRODUCTION

Lung cancer is the leading cause of cancer‐related deaths worldwide.^1^ Recently, some types of molecular‐targeted therapy and angiogenesis inhibitors have been successfully introduced as lung cancer treatments. Epidermal growth factor receptor (EGFR)‐tyrosine kinase inhibitors (TKIs) have been shown to be effective in the treatment of non‐small cell lung cancer (NSCLC) in patients with mutant *EGFR*.^2^, ^3^, ^4^, ^5^ Although EGFR‐TKIs may lead to initial clinical benefits in most patients with *EGFR*‐mutated NSCLC, these patients develop acquired resistance to various EGFR‐TKIs. Therefore, novel therapeutic strategies after resistance to EGFR inhibitors are still needed to improve the prognosis of patients with *EGFR*‐driven lung cancers.

Recently, programmed cell death protein 1 (PD‐1)/programmed death ligand 1 (PD‐L1) checkpoint inhibitors are promising alternative treatments for NSCLC. Of them, nivolumab, pembrolizumab, and atezolizumab have been approved in the United States, Japan, and other countries for the treatment of patients with metastatic NSCLC based on some phase III trials that showed the superior outcomes of PD‐1/PD‐L1 checkpoint inhibitors compared with standard systemic chemotherapy in patients with NSCLC.^6^, ^7^, ^8^, ^9^, ^10^ Several mechanisms for poor responses to immune checkpoint inhibitors (ICIs) have been reported, such as a lower tumor mutation burden and an uninflamed and immunosuppressive tumor microenvironment.^11^ A recent retrospective study showed that subgroups with oncogenic driver mutations, including *EGFR* and anaplastic lymphoma kinase (*ALK*), tend to show a reduced response to PD‐1/PD‐L1 inhibitors regarding objective response rates and progression‐free survival (PFS) when compared with wild‐type *EGFR* and *ALK*‐negative/unknown subgroups among patients with NSCLC.^12^ To date, little is known about the subpopulation of patients with NSCLC with *EGFR* mutations who exhibit clinical outcomes upon receiving ICI treatments. Therefore, to identify eligible patients to treat with ICIs, we retrospectively analyzed the correlations between clinical features and the efficacy of ICIs in patients with *EGFR* mutations.

## 2 MATERIALS AND METHODS

### 2.1 Patients

We enrolled 27 patients with *EGFR*‐activating mutations who were diagnosed with NSCLC, and treated with EGFR‐TKIs and ICIs at 6 different institutions in Japan (University Hospital Kyoto Prefectural University of Medicine, Japanese Red Cross Kyoto Daiichi Hospital, Japanese Red Cross Kyoto Daini Hospital, Uji‐Tokushukai Medical Center, Fukuchiyama City Hospital, and Niigata University Medical and Dental Hospital) between February 2016 and May 2018, regardless of receiving any previous cytotoxic chemotherapy‐containing treatment. We obtained the patients’ clinical data from retrospective medical records, as follows: age, sex, histological subtype, the levels of PD‐L1 expression in tumors, EGFR mutation status at baseline, with or without the emergence of EGFR‐T790M mutation, disease stage, Eastern Cooperative Oncology Group PS, smoking status, the progression‐free survival (PFS), the time to treatment failure (TTF), response rate, disease control rate of patients on initial EGFR‐TKI, and ICI treatments based on the Response Evaluation Criteria in Solid Tumors version 1.1 (RECIST). This study protocol was approved by the ethics committees of each hospital. The tumor–node–metastasis (TNM) stage was classified using version 7 of the TNM stage classification system.

### 2.2 Tumor genomic analysis

*EGFR* mutations were detected using one of the following methods: the peptide nucleic acid–locked nucleic acid clamp (LSI Medience, Tokyo, Japan), Cycleave PCR (Takara bio, Kusatsu, Japan), or Cobas *EGFR* mutation test (Roche Molecular Systems, Pleasanton, CA), with sequencing of exons 18‐21 being performed at commercial clinical laboratories (SRL, Inc and BML, Inc, Tokyo, Japan).

### 2.3 Tumor PD‐L1 analysis

PD‐L1 expression was analyzed at SRL, Inc with the PD‐L1 IHC 22C3 pharmDx assay or 28‐8 pharmDx assay (Agilent Technologies, Santa Clara, CA). The PD‐L1 tumor proportion score (TPS) was calculated as the percentage of at least 100 viable tumor cells for complete or partial membrane staining. The pathologists of the commercial vendor provided the TPS interpretation.

### 2.4 Immunotherapy

The anti‐PD‐1 antibodies administered were nivolumab and pembrolizumab. Nivolumab and pembrolizumab were intravenously administered at doses of 3 mg/kg every 2 weeks and 1200 mg every 3 weeks, respectively. In general, these treatments continued until disease progression, intolerable toxicity, or patient refusal was encountered.

### 2.5 Statistical analysis

Cox proportional hazards models were used, considering age, sex, PS, smoking history, histological type, best response to initial EGFR‐TKIs, metastatic lesions, staging, regimen of ICIs, status of *EGFR* mutation, *EGFR*‐T790M mutation, levels of PD‐L1 expression in tumors, levels of serum albumin, and neutrophil/lymphocyte ratios (NLRs). To analyze the TTF and PFS, times to events were estimated using the Kaplan–Meier method and compared using the log‐rank test. The TTF and PFS were censored at the date of the last visit for patients who remained alive without any documented disease progression. The tumor response was evaluated in accordance with the RECIST, version 1.1. All statistical analyses were performed using Prism (version 7.02; GraphPad Software Inc, CA). All *p* values less than 0.05 were considered statistically significant.

## 3 RESULTS

### 3.1 Patient characteristics

A total of 27 patients with NSCLC who received ICIs, as well as EGFR‐TKIs, which were treated more than one compound, between February 2016 and April 2018 at 6 institutions in Japan were included. Of them, 8 (30%) patients were male and 20 (74%) were never‐smokers, and the median age of all patients was 67 years (range, 37‐82 years). The histological subtypes were adenocarcinoma in 26 patients (96%) and large cell neuroendocrine carcinoma in 1 patient (4%). Twenty‐three patients (85%) had a performance status of 0 or 1. The sites of metastatic disease were the bone, brain, and liver in 12 (44%), 11 (41%), and 4 (15%) patients, respectively. Eighteen patients (67%) had stage IV disease and 9 patients (33%) exhibited recurrence. Twenty‐one (78%) and 6 (22%) patients were administered nivolumab and pembrolizumab, respectively. *EGFR* mutations at baseline were detected as follows: 8 patients harbored a deletion in exon 19, 12 patients harbored an L858R missense mutation in exon 21, 4 patients harbored a G719X mutation in exon 18, and 3 patients harbored an insertion mutation in exon 20. *EGFR*‐T790M mutations after developing resistance to initial EGFR‐TKI treatment were detected in 8 patients, and were not detected in 19 patients (Table 1).

**Table 1:** Patients’ characteristics

Patient characteristics	No. of patients (N = 27)
	N (%)
Age	
Median (Range)	67.0 (37.0‐82.0)
Sex	
Male	8 (29.6)
Female	19 (70.4)
PS	
0/1	23 (85.2)
2	4 (14.8)
Histology	
Adenocarcinoma	26 (96.3)
LCNEC	1 (3.7)
Smoking status	
Never‐smoker	20 (74.1)
Smoker	7 (25.9)
Best response to EGFR‐TKIs	
CR/PR	14 (51.9)
SD	8 (29.6)
PD	1 (3.7)
NE	4 (14.8)
Sites of metastatic disease	
Bone	12 (44.4)
Brain	11 (40.7)
Liver	4 (14.8)
Stage	
IV	18 (66.7)
Postoperative recurrence	9 (33.3)
ICI treatment	
Nivolumab	21 (77.8)
Pembrolizumab	6 (22.2)
EGFR mutations	
EGFR Ex19del	8 (29.6)
EGFR L858R	12 (44.4)
EGFR G719X	4 (14.8)
EGFR exon20 ins	3 (11.1)
Best overall response for ICIs	
CR/PR	6 (22.2)
SD	5 (18.5)
PD	13 (48.1)
NE	3 (11.1)
PD‐L1 TPS	
TPS ≧50%	6 (22.2)
TPS 1～49%	5 (18.5)
TPS <1%	6 (22.2)
NE	10 (37.0)

### 3.2 Association between clinical features and outcomes for patients with *EGFR*‐mutated NSCLC who were treated with ICIs

**Figure 1:**
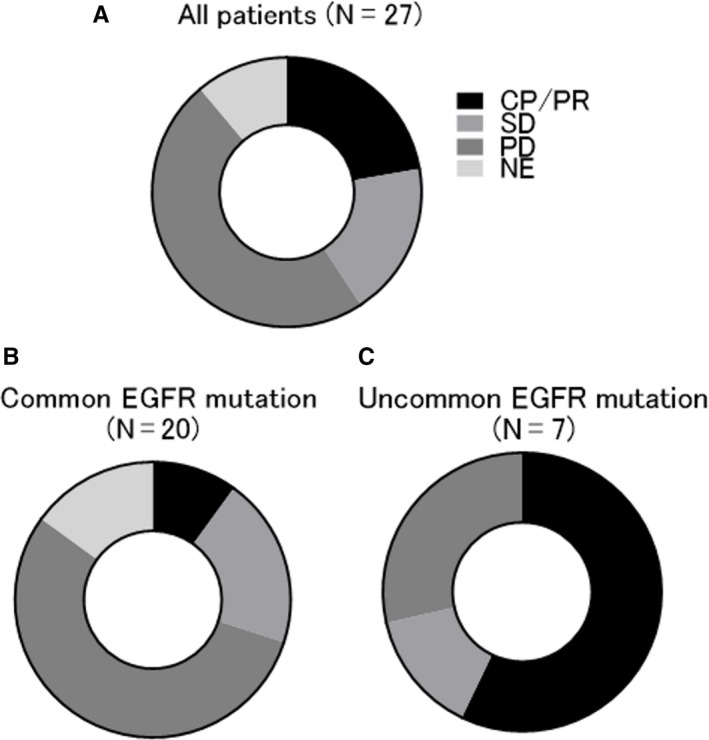
Frequency of best overall response to immune checkpoint inhibitors (ICIs) after acquired resistance to EGFR‐TKI treatment in patients with *EGFR*‐mutated NSCLC. Frequency of best overall response to ICIs for all patients (N = 27) (A), patients with common *EGFR* mutations (N = 20) (B), and patients with uncommon *EGFR* mutations (N = 7) (C) are shown in the pie chart. ICI, immune checkpoint inhibitor; EGFR, epidermal growth factor receptor; TKI, tyrosine kinase inhibitor; NSCLC, non‐small cell lung cancer

**Figure 2:**
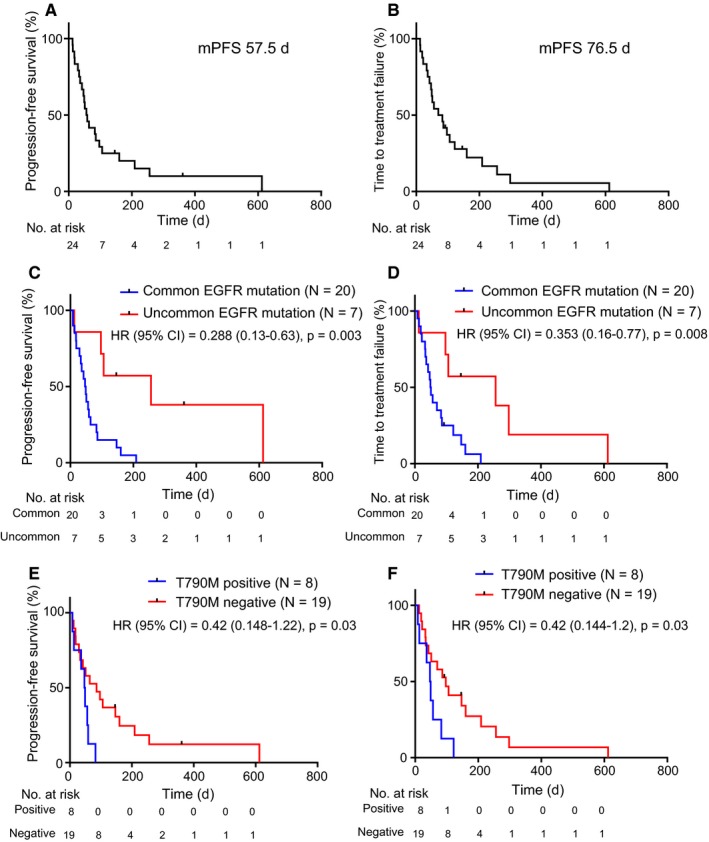
Kaplan‐Meier curves for PFS and TTF in patients with *EGFR*‐mutated NSCLC treated with immune checkpoint inhibitors after acquired resistance to EGFR‐TKI treatment. (A, B) PFS (A) and TTF (B) curves for all patients (N = 27), and (C, D) PFS (C) and TTF (D) curves for patients with common (N = 20) and uncommon (N = 7) *EGFR* mutations. (E, F) PFS (E) and TTF (F) curves for patients with T790M‐positive (N = 7) and T790M‐negative (N = 17) *EGFR* mutations. Column signs denote censoring. PFS, progression‐free survival; TTF, time to treatment failure; EGFR, epidermal growth factor receptor; TKI, tyrosine kinase inhibitor; NSCLC, non‐small cell lung cancer

We showed that a small proportion of patients with lung adenocarcinoma with *EGFR* mutations exhibited favorable clinical benefits when treated with ICIs, nivolumab and pembrolizumab. Of 27 patients with NSCLC with *EGFR* mutations, no patients achieved complete response (CR; 0%), 6 achieved partial response (PR; 22.2%), 5 achieved stable disease (18.5%), 13 achieved progressive disease (48.1%), and 3 were unevaluable (11.1%) when treated with ICIs, which was indicated in a response rate of 22% and disease control rate of 41% (Figure 1A). The median PFS was 57.5 days (8‐612 days) and median TTF was 76.5 days (8‐612 days) (Figure 2A,B).

**Figure 3:**
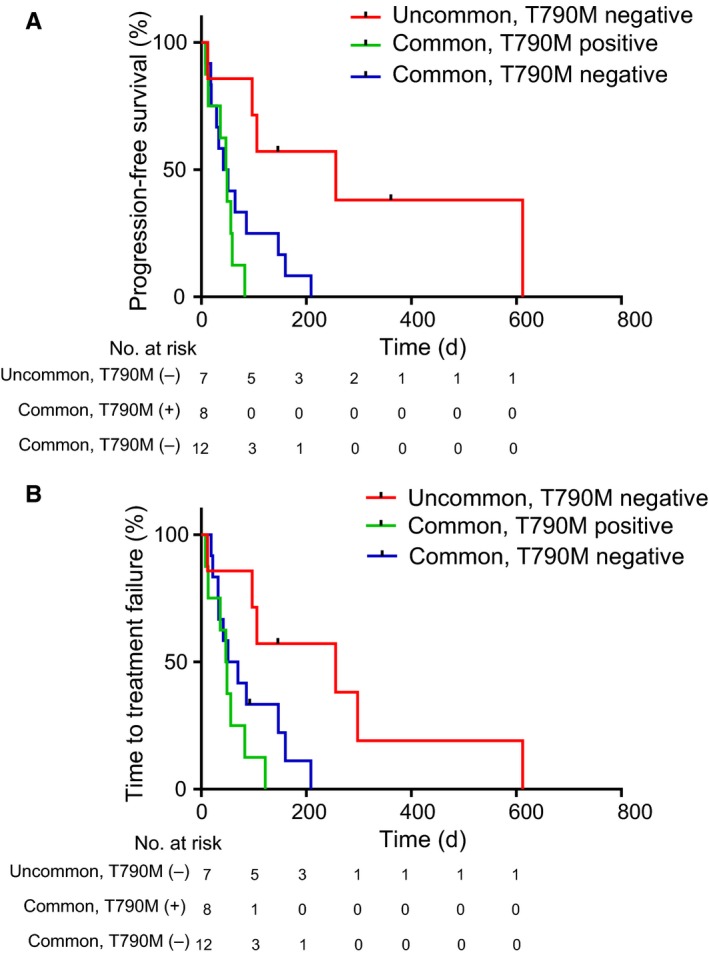
Kaplan–Meier curves for progression‐free survival (PFS) and time to treatment failure (TTF) following treatment with immune checkpoint inhibitors according to *EGFR* mutation status and/or acquired T790M mutations in patients with *EGFR*‐mutated NSCLC. (A, B) PFS (A) and TTF (B) curves for uncommon *EGFR* mutation plus T790M‐positive (red line), common *EGFR* mutation plus T790M ‐positive (green line), and common *EGFR* mutation plus T790M‐negative (blue line) (N = 27) NSCLC. Column signs denote censoring. PFS, progression‐free survival; TTF, time to treatment failure; EGFR, epidermal growth factor receptor; NSCLC, non‐small cell lung cancer

To assess whether the clinical features might be a determinant of ICI efficacy in patients with *EGFR* mutations, we next investigated the association between some clinical parameters and the responders to ICI treatment, which were defined as the CR and PR cases based on the evaluation of RECIST criteria. Of various clinical parameters, only patients with uncommon *EGFR* mutations, including G719X in exon 18 and insertion in exon 20, significantly correlated with responding to ICIs, compared to those with common *EGFR* mutations (hazard ratio of 0.047 with 95% confidence interval of 0.004‐0.557, *P* = 0.015) (Figure 1B,C, Table 2). Although the PD‐L1 TPS in tumors, smoking status, and location of metastatic lesions were known to be predictive factors for therapeutic effects of ICI treatment in NSCLC with wild‐type *EGFR*, there were no significant differences in this study. We next evaluated the correlation between the disease control rate following ICI administration and patient factors, including *EGFR* mutation status and the detection of T790M mutations, that were demonstrated to be relatively promising predictors in the analysis of ICI responders, as shown in Table 2. Although these factors tend to be adequate candidates for prediction, there was no significant correlation with the disease control rate following ICI administration (Supplementary Table S1). We next evaluated the correlation between patient profiles and clinical outcomes of ICI treatment, such as PFS and TTF. Of them, patients with uncommon *EGFR* mutations had significantly better PFS and TTF compared with those in patients with common *EGFR* mutations (256 days vs 50 days, 256 days vs 48 days; hazard ratios of 0.288 and 0.353 with 95% confidence intervals of 0.13‐0.63 and 0.16‐0.77; *P* = 0.003 and 0.008; respectively) (Figure 2C, 2D). In addition, patients with T790M mutations when treated with ICIs had significantly better PFS and TTF compared with those in patients without T790M mutations (86 days vs 48 days, 97 days vs 48 days; hazard ratios of 0.42 and 0.42 with 95% confidence intervals of 0.148‐1.22 and 0.144‐1.2; *P* = 0.03 and 0.03; respectively) (Figure 2E,F). Finally, we validated two promising predictive factors: uncommon *EGFR* mutations and the absence of T790M mutations. T790M mutations were not detected in our analysis in all 7 patients with uncommon *EGFR* mutations. In the cases of common *EGFR* mutations, T790M mutations were detected in 8 patients and but not in 12 patients. These 3 groups (uncommon *EGFR* mutation plus T790M mutation‐positive, common *EGFR* mutation plus T790M mutation‐positive, and common *EGFR* mutation plus T790M mutation‐negative) were statistically different according to PFS and TTF following ICI treatment (*P* = 0.006 and *P* = 0.012, respectively) (Figure 3). These results showed that uncommon *EGFR* mutations in the absence of T790M mutations in patients might be a most potent predictor to detect the responders of ICIs among patients with *EGFR*‐mutated NSCLC.

**Table 2:** Predictive factors according to the response to immune checkpoint inhibitors based on single‐variable analysis

Characteristic	Patients (N = 24)	ICI Response		Odds ratio	*p *value
		Responder (N = 6)	non‐Responder (N = 18)	(95% CI)	
Age (year)					
<70	17	4	13	0.769	>0.999
≧70	7	2	5	(0.11~5.10)	
Gender					
Male	8	2	6	1	>0.999
Female	16	4	12	(0.16~6.50)	
PS					
0/1	22	6	16		
2	2	0	2		
Smoking status					
Never‐smoker	17	4	13	0.769	>0.999
Smoker	7	2	5	(0.11~5.10)	
Best response to EGFR‐TKIs					
CR/PR	11	2	9	0.5	0.649
SD+PD+NE	13	4	9	(0.08~3.04)	
Sites of CNS metastasis					
Present	9	3	6	2	0.635
Absent	15	3	12	(0.37~10.42)	
Sites of Bone metastasis					
Present	11	3	8	1.25	>0.999
Absent	13	3	10	(0.24~6.49)	
Sites of Liver metastasis					
Present	2	1	1	3.4	0.446
Absent	22	5	17	(0.15~67.66)	
Stage					
IV	16	3	13	0.385	0.362
Postoperative recurrence	8	3	5	(0.073~2.18)	
ICI treatment					
Nivolumab	18	5	13	1.92	>0.999
Pembrolizumab	6	1	5	(0.21~26.71)	
EGFR mutation					
Common mutation	17	1	16	0.047	0.015
Uncommon mutation	7	4	3	(0.004~0.557)	
EGFR‐T790M mutation					
Present	7	0	7		0.121
Absent	17	6	9		
PD‐L1 TPS					
TPS ≧50%	6	2	4	1.75	0.618
TPS <50%+NE	18	4	14	(0.25~14.64)	
Alb					
<3.5 mg/dl	8	2	6	1	>0.999
≧3.5 mg/dl	16	4	12	(0.16~6.53)	
NLR					
<4.0	17	6	11		0.13
≧4.0	7	0	7		

CI, confidence interval.

## 4 DISCUSSION

Many promising drugs have been developed for NSCLC, such as molecular‐targeted therapies for *EGFR* and immunotherapy. To date, the effectiveness of ICIs has been reported to be associated with various biomarkers, such as PD‐L1 overexpression in tumors, tumor mutation burden, and smoking exposure in patients with NSCLC.^13^, ^14^ Of them, the frequency of a PD‐L1 TPS of ≥50% is lower in patients with *EGFR*‐mutated or *ALK*‐rearranged NSCLC compared with that in those negative for these genetic changes.^8^, ^15^, ^16^ In addition, the efficacy of PD‐1/PD‐L1 inhibitors is less related to PD‐L1 expression in *EGFR*‐mutated lung cancer.^12^ A meta‐analysis of 3 randomized trials demonstrated that ICIs are less sensitive than docetaxel in subsets of *EGFR*‐mutated advanced NSCLC.^17^ Thus, patients with *EGFR*‐mutated NSCLC generally are unlikely to be ideal candidates to receive ICI treatment. Meanwhile, preclinical studies have shown that activation of *EGFR* signaling pathways involved in the production of PD‐L1 expression in NSCLC cells and anti‐PD‐1 antibodies are effective in mouse models with *EGFR* mutant‐driven tumors.^18^, ^19^, ^20^ In fact, 2 patients with *EGFR* mutations survived for more than 5 years in a phase 1 study of nivolumab treatment. Interestingly, uncommon EGFR mutations, exon20 insertion and G719A, but not common mutations, were detected in these patients.^21^ More recently, a phase III study demonstrated that patients with *EGFR* mutations who were pretreated with EGFR‐TKIs showed superior PFS upon receiving combination therapy of anti‐PD‐L1 antibody atezolizumab plus platinum‐based chemotherapy, compared with that upon receiving chemotherapy alone.^22^ However, the subpopulation of patients with *EGFR*‐mutated tumors who are ideal candidates to receive immunotherapy is still unclear. Therefore, in this retrospective study, we focused on screening for efficacy cases with ICIs among patients with EGFR‐mutated NSCLC.

Liver metastases are known as a poor prognostic factor in patients with lung cancer, regardless of various histologic types,^23^, ^24^ and the presence of liver metastases in patients with NSCLC is associated with shorter PFS and tends to reduce effectivity to PD‐1 inhibition compared with those in patients without liver metastases.^25^ General conditions, including malnutrition, are important factors to consider for successful administration of systemic treatments. In our study, neither metastatic lesions, serum levels of albumin, or PS had an impact on the sensitivity to PD‐1 inhibitors in *EGFR*‐mutated NSCLC. In addition, the serum NLR was not correlated with the outcomes of ICI treatment in *EGFR*‐mutated NSCLC, although the serum NLR was reported to be a potent biomarker according to the benefit of ICI treatments in patients with advanced‐stage cancer.^26^

A previous study reported that a higher Brinkman index, defined as the number of cigarettes smoked per day times the smoking years, greater than 600 may be one of the predictive factors for the efficacy of nivolumab in patients with NSCLC with *EGFR* mutations [10]. Our results indicated that patients with uncommon *EGFR* mutations, G719X and exon 20 insertions, had significantly better response, PFS, and TTF than those in patients with common *EGFR* mutations, such as exon 19 deletions and L858R in exon 21, consistent with findings reported previously.^27^ These findings suggested that patients with uncommon *EGFR* mutations might associate with more specific characteristics of smokers than patients with common *EGFR* mutations, although our study could not indicate the significant relationship between smoking history and the efficacy of ICIs.^28^ As it was reported as a controversial finding, smoking history might be an inadequate and confounding factor in the outcomes of ICIs in *EGFR*‐mutated NSCLC. A previous report showed the negative correlation between the detection of T790M mutations and outcomes of ICI treatment,^29^ which was consistent with our observations that the detection of T790M mutations inversely predicted the PFS and TTF following treatment with ICIs. The *EGFR*‐T790M, the gatekeeper mutation, is the most common mechanism of acquired resistance and is detectable in approximately 50% of patients who develop acquired resistance to first‐ and second‐generation EGFR‐TKIs.^30^, ^31^ The tumors with *EGFR*‐T790M mutations showed low mutation burden in next‐generation sequencing analysis, suggested that these tumors were likely to exhibit reduced responses to ICIs. However, our findings showed that all patients without EGFR‐T790M mutations include those with uncommon EGFR mutations that are most promising predictors in analyzing ICI responders. In addition, patients with common EGFR mutations poorly responded to immunotherapy, independent of *EGFR*‐T790M mutations. Therefore, further investigations are needed to determine the important roles of *EGFR*‐T790M mutations on the detection of responders to immunotherapy. Importantly, this is the first report to show that uncommon *EGFR* mutations without T790M mutations when initiating treatment with ICIs is a promising predictive factor to identify responders of ICIs among patients with *EGFR*‐mutated NSCLC.

This study has several limitations; first, it included a small sample size and was a retrospective study. However, previous retrospective observations regarding these associations have been based on similar sample sizes as that included in this study.^27^ Second, this study only recruited a Japanese cohort. Third, we had various biases on patient conditions when ICI treatment was initiated, such as the number of pretreatment regimens and PS of patients. Fourth, we could not validate the cases harboring uncommon *EGFR* mutations with T790M mutations. Further studies are warranted to develop useful biomarkers of PD‐1/PD‐L1 inhibitors in *EGFR*‐mutated NSCLC.

In summary, NSCLC with uncommon *EGFR* mutations and without T790M mutations was found to be positively associated with clinical benefits of ICI treatment and to be an independent positive prognostic factor in patients with NSCLC with *EGFR* mutations. Further studies are warranted to identify responders to ICIs among patients with *EGFR* mutation‐positive NSCLC.

## CONFLICT OF INTEREST

All authors have no conflict of interest to declare.
