# Supplementary material for: Retrospective efficacy analysis of immune checkpoint inhibitors in patients with EGFR‐mutated non‐small cell lung cancer
Source: Cancer Med. 2019 Feb 21;8(4):1521–9. doi: 10.1002/cam4.2037 (PMC6488155; doi:10.1002/cam4.2037)
Supplement: Supplementary file 1 [file CAM4-8-1521-s001.docx]

| **Supplementary Table 1.** |  |  |  |  |  |
| --- | --- | --- | --- | --- | --- |
| **Predictive factors for the disease control by ICIs in EGFR-T790M mutation by the Single-Variable analysis** | | | | | |
|  |  |  |  |  |  |
|  |  | **ICI Response (RECIST criteria)** | | **Odds ratio** |  |
| **Characteristic** | **Patients (N=24)** | ***CR/PR/SD* (N=6)** | ***PD* (N=18)** | **(95% CI)** | ***p* Value** |
| **EGFR mutation** |  |  |  |  |  |
| Common mutation | 17 | 6 | 11 | 0.2182 | 0.182 |
| Uncommon mutation | 7 | 5 | 2 | (0.038 ～ 1.31) |  |
|  |  |  |  |  |  |
| **EGFR-T790M mutation** |  |  |  |  |  |
| Present | 7 | 1 | 6 | 0.1167 | 0.078 |
| Absent | 17 | 10 | 7 | (0.0094 ～ 1.26) |  |
|  |  |  |  |  |  |
|  |  |  |  |  |  |
| Abbreviations; CI, confidence interval | | | | | |
|  |  |  |  |  |  |
